# *In vitro *gene regulatory networks predict *in vivo *function of liver

**DOI:** 10.1186/1752-0509-4-153

**Published:** 2010-11-12

**Authors:** Youping Deng, David R Johnson, Xin Guan, Choo Y Ang, Junmei Ai, Edward J Perkins

**Affiliations:** 1 Rush University Cancer Center, Rush University Medical Center, Chicago, IL 60612, USA; 2 US Army Engineer Research and Development Center, 3909 Halls Ferry Road, Vicksburg, MS 39180, USA; 3 SpecPro Inc., Vicksburg, MS 39180, USA; 4 School of Computing, University of Southern Mississippi, Hattiesburg, MS 39406, USA

## Abstract

**Background:**

Evolution of toxicity testing is predicated upon using *in vitro *cell based systems to rapidly screen and predict how a chemical might cause toxicity to an organ *in vivo*. However, the degree to which we can extend *in vitro *results to *in vivo *activity and possible mechanisms of action remains to be fully addressed.

**Results:**

Here we use the nitroaromatic 2,4,6-trinitrotoluene (TNT) as a model chemical to compare and determine how we might extrapolate from *in vitro *data to *in vivo *effects. We found 341 transcripts differentially expressed in common among *in vitro *and *in vivo *assays in response to TNT. The major functional term corresponding to these transcripts was cell cycle. Similarly modulated common pathways were identified between *in vitro *and *in vivo*. Furthermore, we uncovered the conserved common transcriptional gene regulatory networks between *in vitro *and *in vivo *cellular liver systems that responded to TNT exposure, which mainly contain 2 subnetwork modules: PTTG1 and PIR centered networks. Interestingly, all 7 genes in the PTTG1 module were involved in cell cycle and downregulated by TNT both *in vitro *and *in vivo*.

**Conclusions:**

The results of our investigation of TNT effects on gene expression in liver suggest that gene regulatory networks obtained from an *in vitro *system can predict *in vivo *function and mechanisms. Inhibiting PTTG1 and its targeted cell cyle related genes could be key machanism for TNT induced liver toxicity.

## Background

High-throughput toxicity testing is predicated upon using *in vitro *cell based systems to rapidly screen and predict how a chemical might cause toxicity to an organ *in vivo *[1]. Recent microarray studies have shown that the *in vitro *gene expression profiles in liver slices treated with various compounds could predict the toxicity and pathology observed *in vivo *[2]. However, the degree to which *in vitro *results can be extended to *in vivo *activity and possible mechanisms of action remains to be fully addressed.

Compared with animal models, primary cell cultures have advantages for investigating mechanisms of chemical toxicity. Primary cultured cells, such as hepatocytes, can offer a convenient system that is easily genetically manipulated and can be used to test various throughput screens using different molecular and biochemical methods. Use of primary cell cultures can also reduce cost and mitigate animal welfare concerns inherent in *in vivo *studies [3]. *In vitro *systems have a long history of use in screening new drugs for human diseases such as cancer and in studying cellular and molecular events of different molecules (e.g., pharmaceuticals and xenobiotics) [4,5].

In this study, we used the nitroaromatic 2,4,6 -rinitrotoluene (TNT) as a model chemical to compare and determine how we might extrapolate *in vitro *data to *in vivo *systems. We compared the gene expression profiles of *in vitro *primary liver cells with the gene expression profiles of *in vivo *liver tissue of rats exposed to TNT.

TNT is a munitions compound which is released to the environment as a result of military training activities and manufacturing processes [6,7]. The major toxic effects to rats in response to TNT exposure are methemoglobinemia, anemia, hypercholesterolemia, and hepatomegaly, splenomegaly, and testicular atrophy with their accompanying histologic lesions [8]. Cytotoxic and genotoxic effects are also caused by TNT [9-11]. Our recent toxicity studies in rats found that TNT decreased body weight, increased liver weight, and induced erythrocytosis (Deng et al., unpublished data).

Although a variety of studies have focused on toxicity aspects of TNT, the underlying mechanisms of toxicity induced by this compound are largely unknown. Several laboratories have started to use microarray analysis to understand the molecular mechanisms of invertebrate ecotoxicity. The exposure of the earthworm *Eisenia fetida *with TNT regulates the expression of genes involved in multiple biological processes including muscle contraction, neuronal signaling and growth, ubiquitinylation, fibrinolysis and coagulation, iron and calcium homeostasis, oxygen transport, and immunity [12]. Microarray analysis of TNT exposed *Arabidopsis thaliana *(Arabidopsis) reveals the induced expression of oxophytodienoate reductases (OPRs), the protein responsible for TNT detoxification in Arabidopsis. The same team also found that bifunctional O- and C-glucosyltransferases is involved in combating TNT induced toxicity in Arabidopsis [2,13]. Eisentraeger et al. [14] investigated the mechanism of human liver cell line HepG2 treated with TNT using the toxicology cDNA microarray. They found that the detoxification metabolism in the HepG2 cells by TNT induced genes encoded phase I and phase II enzymes.

In the current study, we examined gene transcriptional responses to short term (24 h and 48 h) TNT exposures in rat in both *in vitro *hepatocytes and *in vivo *liver. We observed that a number of genes were commonly regulated by both *in vitro *and *in vivo *TNT treatments. Moreover, we found commonly affected pathways in both *in vivo *and *in vitro *systems exposed with TNT. Functional analysis indicates that both TNT treatments *in vivo *and *in vitro *impact genes involved in cell cycle, cell growth and cell death signaling, detoxification response, lipid metabolism and immune response, which can reasonably account for the physiological dysfunctions induced by TNT. In addition, we identified conserved gene networks inferred from *in vivo *and *in vitro *networks. Our results suggest that *in vitro *system can predict *in vivo *functions and mechanisms based on gene expression profiles, and gene network is a valuable approach for predicting *in vivo *function using *in vitro *data.

## Results

### Commonly regulated genes by *in vivo *and *in vitro *TNT treatments

To determine if *in vitro *TNT exposure could be used to predict *in vivo *TNT exposure, the first aim of this experiment was to determine if there were common genes differentially regulated by both *in vivo *and *in vitro *TNT treatments. For the *in vivo *experiment, rats were gavaged with TNT at: 0 (control), 4.8, 48, 96 or 192 mg/kg and sacrificed after 24 h or 48 h. RNA was isolated for microarray hybridization. Agilent rat whole genome array (Agilent Technologies Inc., Palo Alto, CA) was employed in the study. Four biological replicates were used for each unique condition as described in the Material and Methods. For the *in vitro *experiments, primary rat hepatocytes (Lonza, Walkersville, MD) were cultured and treated with TNT at 10 ppm or vehicle control for 24 h, after which RNA was isolated for microarray experiments. The *in vivo *and *in vitro *micorarray data have been deposited in the GEO databases with assigned numbers GSE19628 and GSE19662, respectively.

To identify genes differentially expressed *in vivo*, a one-way ANOVA was applied to identify differentiated transcripts across different doses of TNT treatments at each of the 24 h or 48 h time points. A p value cut-off of 0.05 and a 1.5 fold gene transcript change by comparing at least one pair of conditions between different doses including control were used as filters to identify differentially expressed genes. A total of 4875 gene transcripts were significantly and differentially expressed (i.e., upregulated and downregulated) in *in vivo *TNT treatment for all doses and time points.

**Figure 1:**
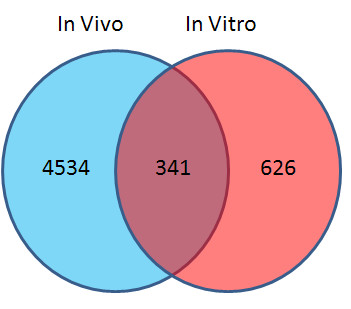
**Commonly significantly regulated transcripts by TNT *in vitro *and *in vivo***. For the *in vivo *experiment, rats were exposed to TNT at various doses: 0 (control), 4.8, 48, 96 or 192 mg/kg for 24 h or 48 h. Subsequently rats were sacrificed and RNAs were isolated for microarray hybridization using a Rat Agilent whole genome array. For *in vitro *experiments, primary cultured rat liver cells were treated with TNT at 10 mg/l or vehicle control, and RNAs were isolated for microarray experiments using the same type of Agilent array as the *in vivo *experiment. Differentiated transcripts were analyzed as described in the Materials and Methods. The commonly regulated transcripts are shown in the intersection part of the Venn diagram.

An un-paired t-test with a p value cut-off of 0.05 and 1.5 fold change filter was used to test for differential expression between controls and TNT treated hepatocytes in the *in vitro *assay yielding 967 differentially expressed transcripts. A Venn diagram shows that there were 341 transcripts that were commonly regulated by both *in vivo *and *in vitro *TNT treatments (Figure 1 & Additional file 1, Table S 1). The common differentiated genes between *in vivo *and *in vitro *exposures suggest that a common mechanism exists between *in vitro *TNT and *in vivo *TNT treated liver systems.

### Similar gene expression pattern shared by commonly regulated *in vivo *and *in vitro *genes

**Figure 2:**
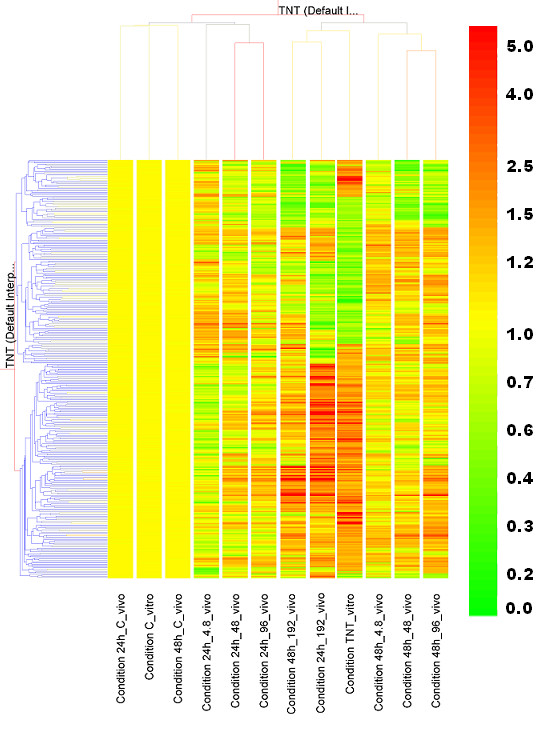
**Hierarchical clustering of experimental conditions**. Experimental conditions were based on averaging samples with the same treatments or controls of both *in vitro *and *in vivo *experiments. Total 12 experimental conditions including 10 *in vivo *and 2 *in vitro *were put together for a Two-Way hierarchical clustering. 341 common transcripts (horizontal axis) were used for clustering across all the conditions (vertical axis). A Pearson correlation algorithm was applied to calculate the distances between transcripts or between conditions. The relative level of gene expression is indicated by the color scale at the right side.

We examined the expression pattern of the 341 common transcripts from *in vivo *and *in vitro *TNT exposures. To reach the goal, each sample was normalized by the mean intensity of a gene of relative control samples, and the samples under the same condition were averaged into one condition. A two-way hierarchical clustering resulted in two cluster dendrograms of the 341 transcripts across 10 *in vivo *conditions and 2 *in vitro *conditions (Figure 2). The controls and 3 lower *in vivo *dose conditions (4.8, 48, and 96 mg/kg) at 24 h were in a cluster, indicating that these doses at 24 h are closer to controls and have less genes affected than other conditions. The remaining *in vivo *and *in vitro *TNT exposed hepatocytes and livers formed the second cluster.

**Figure 3:**
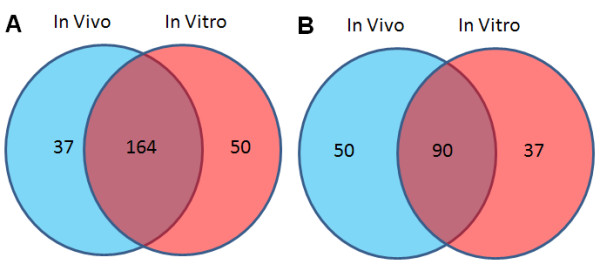
**Comparison of upregulated and downregulated transcripts between *in vivo *and *in vitro *TNT treatments**. Among 341 commonly regulated transcripts regulated by TNT *in vitro *and *in vivo*, commonly upregulated transcripts (A) and downregulated transcripts(B) of these 341 transcripts between *in vivo *TNT treatment at 199 mg/ml for 24 h and *in vitro *TNT treatment are shown by the Venn diagram.

Overall, we found the conditons were more likely to group together according to exposure duration than dose concentration, implying that the time effect is stronger than the dose effect. Interestingly, we found that 3 conditions (*in vivo *TNT treatment at 192 mg/kg for 24 h, *in vivo *TNT treatment at 192 mg/kg for 48 h, and *in vitro *TNT treatment for 24 h) fell into one subgroup. Moreover, we noticed that the gene expression patterns for the 192 mg/kg, 24 h *in vivo *TNT treatment and the 24 h *in vitro *TNT treatment were similar. The genes are upregulated (red pettern) by the *in vitro *treatment, and they were usually upregulated *in vivo *treatment. Most downregulated genes in *in vitro *were also repressed *in vivo*. Since these two conditions behaved closely, we directly compared commonly upregulated and downregulated genes based on these two conditions. Out of these 341 transcripts, 201 transcripts were upregulated by the *in vivo *TNT treatment, and 214 transcripts were upregulated by the *in vitro *TNT treatment. One hundred sixty-four transcripts (77% of the *in vitro *transcripts) were commonly upregulated under both conditions (Figure 3A). One hundred forty *in vivo *TNT transcripts and 127 *in vitro *TNT transcripts were downregulated. Furthermore, 90 transcripts (71% of the 127 *in vitro *transcripts) were reduced by both conditions (Figure 3B). Another common phenomenon was that both TNT exposed liver systems had more upregulated than downregulated genes. Our results demonstrate that TNT treatment in *in vivo *and *in vitro *liver systems results in genes regulated in the same direction, providing more evidence that *in vitro *hepatocytes act as a suitable surrogate for *in vivo *liver exposures.

### Functional analysis of commonly regulated genes

To understand the functions of the commonly regulated genes, we identified most significantly functional categories using the Ingenuity function analysis tool (Ingenuity Systems, Inc., Redwood City, CA). These functional groups mainly included cell cycle, carbohydrate metabolism, molecular transport, cell growth and proliferation, cell death, DNA replication, recombination and repair, lipid metabolism, cellular assembly and organization, and immune response (Table 1). The most significant functional category regulated by TNT was cell cycle, which possessed 31 genes, of which, 18 genes were commonly downregulated by *in vivo *and *in vitro *TNT exposures. The most heavily downregulated genes involved in cell cycle by both *in vivo *and *in vitro *TNT treatments included LGALS1, CYP26B1, and PTTG1. Highly induced genes in this cell cycle category included ADM, DUSP13, and PPARG. More genes participating in cell cycle were downregulated, indicating that TNT appears to interfere with cell cycle progress.

**Table 1:** Significant functional categories based on genes commonly regulated by TNT *in vivo *and *in vitro*

Category	P-value	Downregulated genes*	Upregulated genes*
Cell Cycle	5.1E-07-1E-02	ANGPTL2, LGALS1, CYP26B1, COL1A1, CCND2, CKAP2, CDKN3, KIF20A, CXCL12, ECT2, CCNB1, RARB, PTTG1, LZTS1, TFDP2, BCL2A1, CDC2, INPP5D	MYC, CCNC, DNM1L, NRG2(includesEG:9542), MAPK1, NRG1, TFRC, PPARG, CREG1, RIOK3, NTRK1, DUSP13, ADM
Carbohydrate Metabolism	1.36E-06-1E-02	GCK, MMP2, INPP5 D, PTTG1, CXCL12, LGALS1	NQO1, ADM, PLA2G7, UGDH, GCLC, PPP1R3C, NTRK1, PPARG, JMJD7, PLA2G4B, ABCC3, UGT1A6, CPT1A, PARD3, NRG1, H6PD, SLC5A3, MYC
Molecular Transport	1.36E-06-1E-02	GCK, PTTG1, CXCL12, LGALS1	MYC, SLC5A3, ABCG5, EIF2S1, GMFB, H6PD, MAPK1, NRG1, PARD3, CPT1A, TFRC, ABCC3, PPARG, AQP8, ABCC4, PPP1R3C, GCLC, ADM, NQO1
Cellular Growth and Proliferation	1.25E-05-1E-02	LGALS1, COL1A1, CCND2, KIF20A, CXCL12, CCNB1, RARB, PTTG1, LZTS1, HSD11B2, DLC1, COL1A2, BCL2A1, CDC2, INPP5 D, MMP2	MYC, DNAJB6, NRG2 (includes EG:9542), CES2 (includes EG:8824), MAFF, CDH4 (includesEG:1002), UGT2B17, MAPK1, NRG1, TRIM35, TFRC, PPARG, CXCL2, ALDH1L1, CREG1, GSTP1, HMGCR, NTRK1, CDA, PHLDA1, PFN2, GRIN2C, ADM, CYP1A1
Cell Death	3.45E-05-1E-02	LGALS1, CYP26B1, CCND2, CKAP2, CXCL12, CCNB1, RARB, PTTG1, HSD11B2, DLC1, BCL2A1, CDC2, INPP5 D, MMP2	MYC, DNAJB6, EIF2S1, DNM1L, NRG2(includes EG:9542), GMFB, HTATIP2, MAPK1, NRG1, TRIM35, TFRC, EPHX1, PPARG, CXCL2, SQSTM1, CYP2F1, GSTP1, GSR, NTRK1, ABCC4, PHLDA1, GCLC, PLA2G7, TXNRD1, NR1I3, GRIN2C, ADM, NQO1, NCF2
DNA Replication, Recombination, and Repair	1.52E-04-1E-02	LGALS1, CCND2, CXCL12, ECT2, CCNB1, PTTG1, MMP2	MYC, NRG2 (includes EG:9542), PDE5A, NRG1, PPARG, RIOK3, GSTP1, GCLC, NR1I3, ADM, AMPD3, NQO1
Lipid Metabolism	2.6E-04-1E-02	GCK, INPP5 D, HSD11B2, CXCL12, PNPLA3, LGALS1	CYP1A1, ADM, PLA2G7, PPP1R3C, UGT2B7, NTRK1, ACOT4, GSTP1, AQP8, PPARG, JMJD7-PLA2G4B, ABCC3, RDH16, CPT1A, MAPK1, H6PD, UGT2B17, CYP3A43, CYP2C18, ABCG5, MYC
Cellular Assembly and Organization	5.88E-04-1E-02	LGALS1, COL1A1, ECT2, CCNB1, PTTG1, COL1A2, CDC2, KRT20, INPP5D	MYC, DNAJB6, DNM1L, EPB41, NRG1, RIOK3, PFN2, ADM
Immune Cell Trafficking	3.58E-03-1E-02	COL1A1, CYTIP, CXCL12	CXCL2
Humoral Immune Response	5.57E-03-1E-02	CXCL12, BCL2A1, NPP5D	MYC, NTRK1

* Full names of the genes are listed in Additional file 1, Table S 1.

Other commonly significantly affected functional terms were cell growth and proliferation (40 genes), cell death (19 genes), DNA replication (19 genes), and recombination and repair (19 genes). Interestingly, there were 10 genes that were repeatedly present in all four functional terms: ADM, CCNB1, CCND2, CXCL12, LGALS, MYC, NRG1, NRG2, PPARG, and PTTG1. In addition, these genes were consistently regulated by both *in vivo *TNT and *in vitro *TNT in the same directions. The expression of CCNB1, CCND2, PTTG1, CXC12, and LGALS1 was decreased by TNT, and the expression of ADM, MYC, NGR1, NGR2, and PPARG was elevated by TNT.

There were 23 commonly regulated genes that were involved in molecular transport (Table 1). Nineteen of the 23 genes (83%) were upregulated, and only 4 of them were downregulated by both *in vitro *and *in vivo *TNT treatments. Some significant upregulated genes included NQO1, ADM, ABCC3 and ABCC4. Our results suggest that TNT can enhance molecular transport both *in vivo *and *in vitro *liver systems.

Twenty-three genes contributing to carbohydrate metabolism were commonly regulated by both *in vivo *and *in vitro *TNT treatments. Among them, only 6 genes were repressed and 17 genes were induced. The expression of 27 genes participating in lipid metabolism was significantly changed in response to both *in vivo *and *in vitro *TNT exposures. Similar to carbohydrate metabolism, we saw more upregulated genes (21) than downregulated genes (6) in this category. Several cytochrome P450 family members such as CYP1A1, CYP3A43 and CYP2C18 were in the upregulated gene list. Interestingly, 10 genes (MYC, PPARG, PPP1R3C, CXCL12, GCK, ABCC3, ADM, CPT1A, H6PD, and LGALS1) belong to the three functional categories: molecular transport, carbohydrate metabolism and lipid metabolism. Three of these 10 genes (LGALS1, GCK, CXCL12) were downregulated and the other 7 genes were upregulated by both *in vivo *and *in vitro *TNT treatments.

There were 17 commonly regulated genes that were involved in cellular assembly and organization. The expression of 10 genes was decreased, while expression of 7 genes were increased. Eight genes played a role in immune response, 5 of which were downregulated and 3 genes upregulated. More immune response genes were downregulated, indicating that TNT may interfere with normal immune function to induce its toxicity.

Overall, some commonly regulated genes were highly represented. For instance, the gene MYC falls into all the functional categories listed in Table 2. CXCL12 belongs to all the functional groups except cellular assembly and organization. ADM and LGALS1 genes PPARG were absent in only two functional categories, cellular assembly and organization, and immune response.

**Table 2:** Commonly regulated canonical pathways based on *in vivo*, *in vitro *and common gene lists regulated by TNT

Pathway	-Log(P-value) *in vivo*	-Log(P-value) *in vitro*	-Log(P-value) *in vivo *and *in vitro*
Metabolism of Xenobiotics by Cytochrome P450	7.26	4.77	5.82
NRF2-mediated Oxidative Stress Response	6.17	6.05	5.31
LPS/IL-1 Mediated Inhibition of RXR Function	5.66	6.29	3.48
Glutathione Metabolism	8.31	3.33	3.14
Xenobiotic Metabolism Signaling	5.05	5.14	4.4
Pentose and Glucuronate Interconversions	4.63	3.67	4.37
Aryl Hydrocarbon Receptor Signaling	3.82	3.84	3.93
PXR/RXR Activation	3.28	4.34	1.84
Pyruvate Metabolism	4.54	2.15	1.25
Galactose Metabolism	4.11	1.44	1.73
Fructose and Mannose Metabolism	4.19	1.33	1.65
Retinol Metabolism	1.98	1.97	2.59
Biosynthesis of Steroids	1.83	1.56	2.28
Fatty Acid Metabolism	2.3	1.87	1.48
Starch and Sucrose Metabolism	1.11	1.48	2.24
Androgen and Estrogen Metabolism	1.31	1.34	1.74

### Pathway analysis

**Figure 4:**
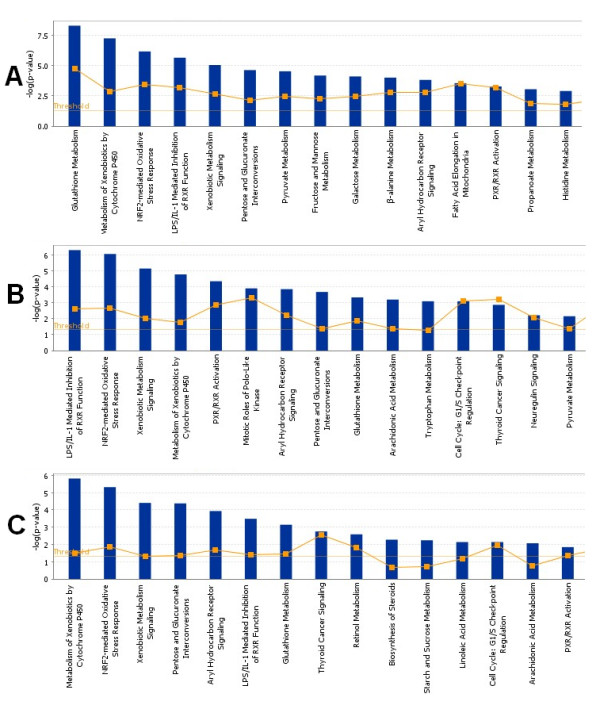
**Top significantly canonical pathways based on *in vivo*, *in vitro *and common gene lists regulated by TNT**. Three separate gene lists resulted from most significantly regulated gene list *in vivo*(A), significantly regulated gene list *in vitro*(B) and commonly regulated gene list between *in vivo *and *in vitro *(C) were used to run the pathway analysis. The bigger the -log(p-value) of a pathway is, the more significantly the pathway is regulated. The threshold lines represent a p value with 0.05. Top 15 most significantly regulated pathways for each list are presented.

**Figure 5:**
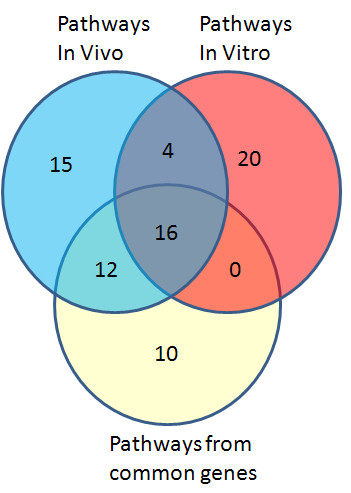
**Comparison of significantly canonical pathways based on *in vivo*, *in vitro *and common gene lists regulated by TNT**. Three separate gene lists resulted from most significantly regulated gene list *in vivo*, significantly regulated gene list *in vitro *and commonly regulated gene list between *in vivo *and *in vitro *were used to run the Ingenuity pathway analysis tool. The overlapped significantly regulated pathways are presented in the Venn diagram. A pathway enrichment p value less than 0.05 was considered as significant.

To further understand the gene function influenced by TNT exposure to *in vivo *and *in vitro *liver cells, we conducted a canonical pathway analysis using the Ingenuity pathway analysis tool. Three separate gene lists were used to run the pathway analysis: the list of genes most significantly regulated by *in vivo *TNT treatment, the list of genes most significantly regulated by *in vitro *TNT exposure, and the list of the 341 commonly regulated genes. In order to identify the most significantly influenced pathways *in vivo*, a more stringent p value (p < 0.002) was applied, which resulted in 1106 significantly regulated transcripts for the *in vivo *TNT experiment. The top significantly impacted pathways from each gene list are depicted in Figure 4. Using a threshold p value of 0.05, a total of 47 pathways were regulated by *in vivo *TNT treatment, 40 pathways were evidently affected by *in vitro *TNT exposure, and 38 significantly pathways were obtained by analyzing the common 341 genes alone (Figure 5).

Twenty commonly regulated pathways were shared by the *in vivo *and *in vitro *pathway lists, which is 50% of the pathways impacted by *in vitro *TNT treatment (Figure 5). The percentage of overlapping pathways was much higher than that of overlapping genes illustrated in Figure 1, indicating that the *in vitro *system perturbed by TNT could reflect more biological truth for an *in vivo *system from a mechanism related pathway view. Sixteen consensus pathways were shared by the three significant pathway lists (Figure 5 and Table 2). The top regulated pathways with relative genes are listed in Table 3. Several gene families that were highly represented in these pathways are related to xenobiotic metabolism and transport. These families were cytochrome P450 (CYP), glutathione S-transferase (GST), UDP glucuronosyltransferase (UGT), aldehyde dehydrogenase 1(ALDH1) and ATP-binding cassette (ABC) families. The regulated genes in the CYP family included CYPA1, CYP2C18, CYP2F1, and CYP3A43. The genes listed in Table 3 belonging to GST family included GST1 and GSTA5. The UGT family included UGT1A6, UGT2B7, and UGT2B17. The regulated gene aldehyde dehydrogenase 1 family, member L1 (ALDH1L1) in the family ALDH1 was involved in many pathways listed in Table 3. The regulated genes in the ABC family included ABCC3, ABCC4, and ABCG5.

**Table 3:** Top common pathways regulated by TNT *in vitro *and in vivo

Canonical Pathways	Common regulated genes*
Metabolism of Xenobiotics by Cytochrome P450	CYP3A43, CYP2F1, GSTP1, CYP1A1, CYP2C18, UGT1A6, UGT2B7, GSTA5, ALDH1L1, UGT2B17, EPHX1
NRF2-mediated Oxidative Stress Response	GSR, AKR7A3, GSTP1, MAPK1, GSTA5, NQO1, GCLC, SQSTM1, DNAJB6, MAFF, TXNRD1, EPHX1
Xenobiotic Metabolism Signaling	GSTP1, CYP1A1, UGT1A6, MAPK1, GSTA5, NQO1, ALDH1L1, GCLC, CES2 (includes EG:8824), UGT2B7, NR1I3, UGT2B17, ABCC3
Aryl Hydrocarbon Receptor Signaling	MYC, GSTP1, CYP1A1, CCND2, MAPK1, GSTA5, NQO1, RARB, ALDH1L1
Pentose and Glucuronate Interconversions	TCAG7.1260, AKR7A3, UGDH, UGT1A6, UGT2B7, UGT2B17
LPS/IL-1 Mediated Inhibition of RXR Function	GSTP1, ABCG5, CPT1A, GSTA5, NR1I3, ALDH1L1, CES2 (includes EG:8824), ABCC3, ABCC4
Glutathione Metabolism	GSR, GSTP1, GSTA5, H6PD, GCLC
PXR/RXR Activation	CPT1A, NR1I3, CES2 (includes EG:8824), ABCC3

* Full names of the genes are listed in Additional file 1, Table S 1

Interestingly, all the genes in Table 3, except for CCND2 and retinoid acid receptor beta (RARB), were commonly upregulated by both *in vivo *and *in vitro *TNT treatments. MAPK1, a critical intracellular signaling protein involved in multiple cellular functions (Table 1) was also presented in multiple pathways such as NRF-2 mediated oxidative stress response, xenobiotic metabolism signaling, and aryl hydrocarbon signaling pathways.

### Inferring *in vitro *and *in vivo *gene networks

**Figure 6:**
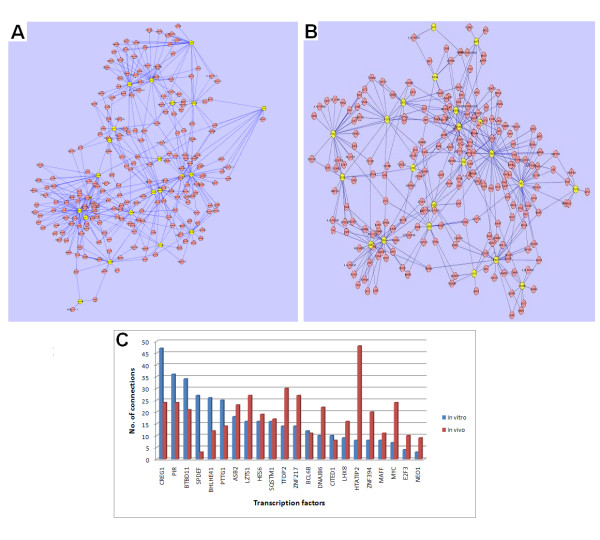
***In vivo *and *in vitro *transcriptional gene network views**. Both gene networks were built using common genes regulated by TNT *in vivo *and *in vitro*. The *in vivo *gene network (A) was constructed using 200 arrays from rat liver tissues treated with one of 5 compounds TNT, 2,4-DNT, 2,6-DNT, 2A-DNT and 4A-DNT or vehicle controls. The *in vitro *gene network (B) was modeled using 531 arrays resulted from liver primary cultured cells treated by one of 105 compounds with relative controls. The Context Likelihood of Relatedness (CLR) algorithm was employed to build both gene networks. Yellow highlighted genes are transcription factors. (C) Number of connections of transcription factors *in vivo *and *in vitro *gene networks. The number of connections of transcription factors *in vivo *and *in vitro *gene networks exhibited in Fig. 6A, B was counted.

Motivated by commonly regulated genes, functional terms and pathways, we then investigated whether a common gene network was regulated by both the *in vitro *and *in vivo *TNT treatments. To achieve this goal, we used a reverse engineering algorithm called Context Likelihood of Relatedness (CLR). CLR is based on mutual information of any pair of genes and is a static network. A static gene network reflects all possible gene interactions for a given gene list. Therefore, the more samples used to construct a gene network using this algorithm make the built network more trustworthy. In order to build *in vivo *gene networks, 199 arrays from rat liver tissues treated with one of 5 compounds TNT, 2-amino-4,6-dinitrotoluene (2A-DNT), 4-amino-2,6-dinitrotoulene (4A-DNT), 2,4-dinitrotoluene (2,4-DNT) and 2,4-dinitrotoluene (2,6-DNT) or vehicle controls were used (S. Meyer, unpublished data). For *in vitro *gene network modeling, 531 arrays resulted from liver primary cultured cells treated by 105 distinctive compounds with relative controls were employed (D. Johnson, unpublished data). Using the gene expression data of the commonly regulated 341 transcripts across the 199 arrays, an *in vivo *gene network was constructed with the CLR algorithm. Figure 6A depicts the network which included 242 nodes and 417 edges. *In vitro *gene network was built using the same 341 genes and the same CLR algorithm, but expression data were generated from the 531 arrays. The gene network from the *in vitro *data had 235 nodes and 352 edges (Figure 6B).

Since transcriptional regulated gene networks are trigged by transcription factors, we then counted the connections of the transcription factors (TFs) in the 341 transcripts for the both *in vivo *and *in vitro *gene networks. Top TFs with most connections in the *in vitro *gene network were cellular repressor of E1A-stimulated genes 1 (CREG1), pirin (iron-binding nuclear protein) (PIR), BTB (POZ) domain containing 11(BTBD11), SAM pointed domain containing ets transcription factor (SPDEF) and basic helix-loop-helix family, member e41 (BHLHE41). TFs with most connections in the *in vivo *gene network included HIV-1 Tat interactive protein 2, 30kDa (HTATIP2), transcription factor Dp-2 (E2F dimerization partner 2) (TFDP2), leucine zipper, putative tumor suppressor 1 (LZTS1), zinc finger protein 217 (ZNF217), PIR, and MYC. The transcription factors that had over 15 connections in the both gene networks included CREG1, PIR, BTBD11, LZTS1, ASB2, HES6, and SQSTM1(Figure 6C). Two TFs, SPDEF and BHLHE41, had at least twice more connections in the *in vitro *gene network than that in the *in vivo *gene network. Seven TFs including HTATIP2, MYC, NEQ1, ZNF394, E2F3, DNAJB6 and TFDP2 possessed over twice as many connections in the *in vivo *gene network than that in the *in vitro *gene network.

### Conserved *in vitro *and *in vivo *gene networks

**Figure 7:**
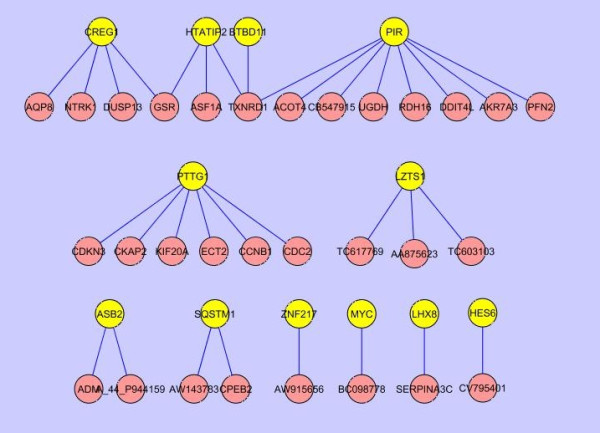
**Conserved sub gene networks between *in vitro *and *in vivo *gene networks**. By comparing *in vivo *and *in vitro *gene networks (shown in Fig. 6), conserved subnetworks that had the same connections in the both networks were achieved. Transcription factors are highlighted as yellow.

By comparing the *in vivo *and *in vitro *gene networks (Figure 6), we identified several conserved sub-networks that were common in both gene networks (Figure 7). The TF PTTG1 centered sub-gene network consisted of 7 genes (PTTG1, CCNB1, CDC2, CDKN3, CKAP2, ECT2, and KIF20A), all of which are involved in cell cycle processes. Moreover, all the genes in the cell cycle sub-network were consistently repressed by both *in vivo *and *in vitro *TNT treatments. The conserved sub-network provides further evidence that the cell cycle process may be impaired in both *in vivo *and *in vitro *systems treated with TNT.

The TFs PIR, BTBD11, HTATIP2, and CREG1 were colocalized in the biggest conserved sub-network (herein called the PBHC network). Eight genes were connected with the TF PIR centered sub-gene network module (Figure 7), and contained highly enriched genes associated with oxidoreductase activity (ACOT4, AKR7A3, RDH16, TXNRD1, and UGDH). AKR7A3 and TXNRD1 also participate in NRF2-mediated oxidative stress pathway. All the genes in this network module were upregulated by both *in vivo *and *in vitro *TNT exposures. This result suggests that oxidative stress is activated by TNT [15].

The TF CREG1 centered sub-network module included 5 genes (Figure 7), 3 of which were also cell cycle related genes (CREG1, NTRK1 and DUSP13). In contrast to PTTG1 centered sub-network, all the genes in the CREG sub-network module were upregulated by both *in vivo *and *in vitro *TNT treatments. Glutathione reductase (GSR), a gene related to oxidoreductase activity, and TXNRD1 provided the connections between the CREG1, HTATIP2, and PIR sub-networks (Figure 7), creating a larger 13 gene sub-network. In addition, GSR also participated in the NRF2-mediated oxidative stress pathway. Indeed, we found that all the genes in the biggest conserved sub-network formed by CREG1, HTATIP2, BTBD11, and PIR mediated modules were consistently upregulated by both *in vivo *and *in vitro *TNT additions.

### Verification of microarray responses using real time QRT-PCR

**Figure 8:**
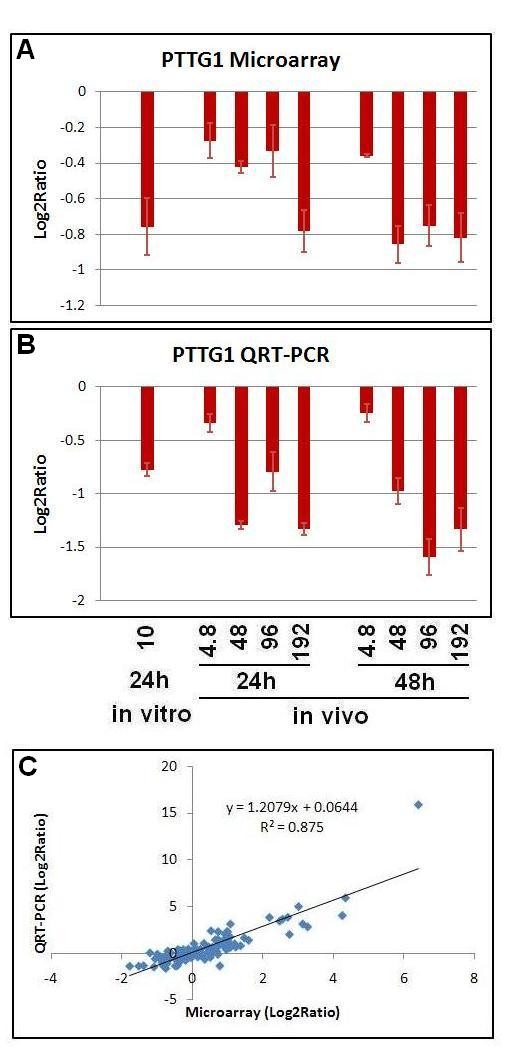
**Quantitative real-time RT-PCR (QRT-PCR) verification of microarray gene expression**. Both QRT-PCR (A) and microarray (B) results of the gene PTTG1(G) were presented. The experimental design was the same as the microarray experiment. The expression value for a given gene was represented as a log2 ratio of ratio of exposed versus respective control RNA. The correlation of total 13 genes across different conditions (n = 156) between the microarray and RT-PCR data was shown on Fig. 8C. Bars represent the standard errors for the average log2 ratios of biological replicates.

To verify the credibility of microarray and gene network modeling results, we selected 13 genes to perform real time quantitative PCR (QRT-PCR) both *in vitro *and *in vivo*. The genes chosen consistently played key roles in functional categories and pathways altered by TNT exposures *in vivo *and *in vivo*. As illustrated in Figure 8, we observed good consistency between microarray and QRT-PCR results. Similar upregulation and downregulation trends were seen with both microarray data and QRT-PCR data for all 13 genes. The overall regression coefficient of the two methods across various conditions was 0.875 (Figure 8C). Our results indicate the microarray data quality is good and our interpretation and analysis should be convincing. Specific comparisons between microarray and QRT-PCR are presented in Additional file 2, Figures S 1A-1F.

For instance, the gene ABCC3 was shown in microarray (Additional file 2, Figure S 1A) to be upregulated at both 24 h and 48 h with a clear dose response to *in vivo *TNT treatment, and was also induced by TNT *in vitro*, and the same pattern was exhibited by QRT-PCR (Additional file 2, Supplementary Figure 1A). In both microarray and QRT-PCR results, the gene AKR7A3 was upregulated at both time points *in vivo *as well as upregulated *in vitro*. Interestingly, AKR7A3 was significantly upregulated at the highest dose (192 mg/kg) *in vivo *at 48 h but not at other lower doses, which was shown consistently by both microarray and QRT-PCR (Additional file 2: Figure S1 A). For the gene CYP1A1, we saw it was upregulated more in response to *in vitro *TNT than *in vivo *TNT treatment, and was induced much more at 24 h than 48 h *in vivo*, which was consistently revealed by both microarray and QRT-PCR results (Additional file 2, Figure S 1C). Three genes PTTG1 (Figure 8), CCNB1 and CCND2 that were in PTTG1 centered subnetwork were consistently shown to be downregulated by TNT *in vivo *and *in vitro*, with both approaches (Additional file 2, Figures S 1B and F). The QRT-PCR results could exactly confirm our two observations obtained from the microarray data: the genes up or downregulated in the same directions *in vitro *and *in vivo*, and a clear dose response *in vivo*.

## Discussion

In this study, we compared gene expression profiles regulated by TNT exposure *in vivo *and *in vitro *from shared gene lists, functional terms, common pathways, and conserved networks. Overall, good consistency existed between *in vitro *and *in vivo *exposures. The Venn diagram (Figure 1) shows that a only small percentage of the *in vivo *transcripts were also regulated in the *in vitro *experiments. Because the regulated gene number *in vivo *is the total number from all TNT treatments including multiple doses and two time points (24 h and 48h), the gene number is much larger than that of *in vitro *exposures. However, the *in vitro *gene number comes from only one dose. The reason we used one dose is that the *in vitro *experiment is a large one with a total of over 105 chemicals and 531 arrays. But if we compare the overlapped transcripts with the *in vitro *transcripts (940), the number of overlapped transcripts is not small, with over one third of the total *in vitro *transcripts. This observation is found more than in overlaps examined in other publications ([16]. Also we would like to emphasize that the gene expression patterns for the 192 mg/kg, 24 h *in vivo *TNT treatment and the 24 h *in vitro *TNT treatment are similar. If we only count the regulated transcript number for 192 mg/kg, 24 h *in vivo *TNT treatment (Figure 2), the number is comparable to that of the 24 h *in vitro *TNT treatment. In addition, *in vivo *and *in vitro *are two different systems, we cannot expect that they are exactly the same. Our purpose is to identify common transcripts between these two systems, thereby to understand the *in vivo *function through the *in vitro *system. From the perspective of functional analysis, both TNT treatments *in vivo *and *in vitro *influence genes involved in cell cycle, cell growth and cell death signaling, detoxification response, lipid metabolism and immune reponse, which can reasonably explain the physiological dysfunctions induced by TNT. We also found conserved sub-networks between inferred networks from *in vivo *and *in vitro *TNT regulated gene expression profiles.

### Cell cycle, cell growth, and cell death signaling

A large number of genes involved in cell cycle, cell growth and cell death were commonly regulated in both *in vitro *and *in vivo *systems perturbed by TNT. The highly represented downregulated genes were CCNB1, CCND2, PTTG1, CXC12, and LGALS1, and the highly represented upregulated genes were ADM, MYC, NRG1, NRG2, and PPARG. The cyclin B1 (CCNB1) gene product complexes with p34(cdc2) to form the maturation-promoting factor (MPF). CCNB1 is expressed predominantly during G2/M phase because of its critical role in cell mitosis [17,18]. The protein encoded by CCND2 belongs to the highly conserved cyclin family, whose members are characterized by a dramatic periodicity in protein abundance through the cell cycle. Cyclins function as regulators of CDK kinases. Different cyclins exhibit distinct expression and degradation patterns which contribute to the temporal coordination of each mitotic event. The CCND2-translated cyclin forms a complex with and functions as a regulatory subunit of CDK4 or CDK6, whose activity is essential for cell cycle G1/S transition. PTTG1 encoded protein is a homolog of yeast securin proteins, which prevent separins from promoting sister chromatid separation. The gene product contains 2 PXXP motifs, which are essential for its transforming and tumorigenic activities, as well as for its stimulation of basic fibroblast growth factor expression [19-21]. Besides the functions of cell cycle and growth, the major role of CXC12 is involved in immune response. The protein encoded by LGALS1 is a member of beta-galactoside-binding protein family implicated in modulating cell-cell and cell-matrix interactions, which may act as an autocrine negative growth factor that regulates cell proliferation [22].

The protein product of the gene Adrenomedullin (ADM) is a multifunctional peptide vasodilator that carries out its functions through calcitonin receptor-like receptor/receptor activity modifying protein-2 and -3 (CLR/RAMP2 and CLR/RAMP3). It can positively enhance cell proliferation [21]. MYC is a well known multifunctional, nuclear phosphoprotein that contributes to cell cycle progression, apoptosis and cellular transformation [23]. It acts as a transcription factor that regulates transcription of specific target genes. It has been implicated as an oncogene to facilitate cell growth and survival. The protein encoded by NRG1 was initially characterized as a 44-kD glycoprotein that interacts with the NEU/ERBB2 receptor tyrosine kinase to increase its phosphorylation on tyrosine residues. This protein is a signaling protein that regulates cell-cell interactions and is involved in the promotion of growth and development of multiple organ systems [24,25]. Neuregulin 2 (NRG2) is a new member of the neuregulin family of growth and differentiation factors. By interacting with the Erbb family of receptors, NRG2 induces the growth and differentiation of epithelial, neuronal, glial, and other cell types [26]. The gene PPARG encodes a member of the peroxisome proliferator-activated receptor (PPAR) subfamily of nuclear receptors. PPARs form heterodimers with retinoid X receptors (RXRs) and these heterodimers regulate transcription of various genes to execute various functions including lipid metabolism and cell growth [27,28].

The gene BCL2A1, encodes a member of the BCL-2 protein family, a well known protein family functioning as anti- and pro-apoptotic regulators[29]. The protein encoded by this BCL2A1 is able to reduce the release of pro-apoptotic cytochrome c from mitochondria and block caspase activation, thereby enhance cell survival. The reduction of the expression of this gene could partially account for the cell damage induced by TNT.

Interestingly, the downregulated genes involved in cell growth and cell death also play a role in the cell cycle process. Therefore, the cell growth and other possible cell death related phenotypes may occur primarily through interrupting cell cycle progression of cells. Recently we found that there was weight loss in rats treated with TNT (data not shown). The possible reason for the body weight loss is that cell growth is reduced by TNT. TNT could inhibit the growth of V79 and TK6 human lymphoblastic cells [30]. It could explain cytotoxic effects caused by TNT [9-11].

### Detoxification response

Our results revealed a couple of pathways that were commonly regulated by both *in vivo *and *in vitro *TNT treatments. From the pathways listed in Table 3, we identified xenobiotic metabolism signaling as the key of all the pathways because all the other pathways are involved in xenobiotic metabolism signaling. Certainly, several phase I and II metabolizing enzymes with their family members that take part in multiple pathways play a pivotal role in the detoxification process. These enzymes mainly cover CYPs, GSTs, UGTs, AKR7A3, ALDHL1, NQO1, and EPHX1. As expected, all these enzymes were consistently elevated by both *in vitro *and *in vivo *TNT treatments.

CYP1A1 has been a hallmark for the treatments of many toxins and carcinogens [31-34]. CYP1A1 was strongly induced in both *in vivo *and *in vitro *liver cells by TNT. Due to its catalytic function, it could play dual roles in the cells in the presence of TNT. Its activation leads to the production of reactive oxygen species (ROS) [35] which induces oxidative stress, lipid peroxidation (LPO), protein modification and denaturation, and DNA damage, and thus induces toxicity. Meanwhile, it may also directly metabolize TNT or its metabolites, resulting in cell survival. Glutathione transferases (GSTs) catalyze the conjunction of reduced glutathione (GSH) to electrophiles and oxidative stress products, thus facilitating their removal [36].

UGTs catalyze the generation of glucuronide conjugates of dihydrodiols to execute their detoxification function [37]. AKR7A3 catalyzes aflatoxin B1 (AFB 1) and suppresses AFB1 dialdehyde metabolite to its corresponding mono and dialcohols. Hence, the activation of AKR7A3 attenuates the toxicity of the AFB1-dialdehyde that reacts with proteins, and thus decreases AFB1-induced toxicity [38].

ALDH1L1 is well recognized as protectors against ROS caused oxidative damage. It metabolizes reactive products of toxic LPO, which include 4-hydroxy-2-nonenal (4-HNE) and malondialdehyde (MDA) [39]. This protective mechanism could explain how ALDH responds to the cellular recovery to TNT exposure.

As a cytosolic flavoenzyme, NQO1 catalyzes the two-electron reduction of diversified substrates [40]. NQO1 is characterized as a detoxification enzyme mainly because of its capability of degenerating quinone substrates to their less toxic hydroquinone metabolites, bypassing the redox-cycling semiquinone radical [41,42]. NQO1 can also transform ubiquinone and vitamin E quinone to their antioxidant forms [42].

EPHX1 is one member of epoxide hydrolases (EPHs) that catalyze the hydrolysis of electrophiles such as epoxides to the less reactive vicinal diols, which can explain the mechanism of epoxide hydrolases as classical detoxifying enzymes. EPH is able to inactivate highly diversified reactive epoxides with different structures, and therefore plays a critical role in the enzymatic defense against adverse effects of xenobiotic compounds [43].

Many upregulated genes involved in xenobiotic metabolism signaling also participate in cell cycle, cell growth, and cell death. Our results suggest that, upon exposure to TNT, cells arrest the cell cycle and increase detoxification and oxidative stress enzymes to remove the xenobiotic and eliminate any cell damage caused by the xenobiotic..

### Lipid metabolism

Several genes participating in lipid metabolism were commonly regulated by both *in vivo *and *in vitro *TNT exposures. Most of these lipid metabolism genes showed elevated expression, while only a small number of genes were repressed. The downregulated genes included LGALS1, PNPLA3, CXCL12, HSD11B2 and INPP5 D. One well known gene, inositol polyphosphate-5-phosphatase, 145kDa (INPP5 D, or SHIP-1), encodes a protein that is largely confined to hematopoietic cells. It is a well characterized inhibitory molecule that is recruited by engagement of the inhibitory Fcγ type IIB receptor in B cells and mast cells or by engagement of Fcε type I or Fcγ type III, cytokine, and growth factor receptors in myeloid cells [44]. Once recruited to the plasma membrane by signaling complexes, its enzymatic activity depletes PtdIns(3,4,5)P_3 _and prevents membrane localization of some PH domain-containing effectors, eventually leading to impaired PI3K-dependent signaling events.

The protein encoded by the gene PNPLA3 is a member of the adiponutrin family complement the hormone sensitive lipase (HSL) as responsible for adipocyte triacylglycerol lipase activity. Mice lacking HSL reveal a lean phenotype and accumulate diglycerides suggesting that HSL is the main enzyme for the second step of lipolysis [45]. LGALS1 has been shown to be involved in many other functions in Table 1, also plays a role in lipid metabolism, by increasing the induction of levels of phosphatidylserine as well as the mobilization of phosphatidylserine [46]. Hydroxysteroid (11-beta) dehydrogenase 2 (HSD11B2) has been reported to contribute to the metabolism of aldosterone, cortisone, glucorticoid and hydrocortisone [47,48]. The downregulated genes involved in lipid metabolism may indicate that TNT may interrupt lipid metabolism to some degree. The lipid metabolism was also impaired in the liver of quail exposed to 2,6-DNT [49]. Meanwhile, we saw many other genes involved in lipid metabolism upregulated by TNT, many of which overlap with xenobiotic signaling genes (Table 3), suggesting that they also function for detoxification by trying to recover normal lipid metabolism.

### Immune response

As described earlier in the results, 5 of 8 genes associated with immune response were downregulated by TNT both *in vivo *and *in vitro*. These 5 genes are CXCL12, COL1A1, BCL2A1, INPP5 D, and CYTIP.

Chemokine (C-X-C motif) ligand 12 (CXCL12 or SDF-1) was strongly downregulated by TNT. It is a ligand for the G-coupled receptor protein chemokine (C-X-C motif) receptor CXCR4. Activation of CXCR4 by CXCL12 is involved in many biological functions such as cell migration, growth, and survival [50].

Besides enhancing cell survival, BCL2A1 is a direct transcription target of NF-κB in response to inflammatory mediators, and is upregulated by different extracellular signals such as granulocyte-macrophage colony-stimulating factor (GM-CSF), CD40, phorbol ester and inflammatory cytokines TNF and IL-1, which suggests a cytoprotective function that is essential for lymphocyte activation, and plays a role in immune response [51].

COL1A1 encodes the pro-alpha 1 chains of type I collagen whose triple helix is composed of two alpha 1 chains and one alpha 2 chain. Type I collagen is a fibril-forming collagen found in most connective tissues and is abundant in bone, cornea, dermis and tendon. Thus this gene is critical for cell aggregation, migration, proliferation, binding, adhesion and growth [52]. This gene carries out its immune response by primarily affecting transmigration of T lymphocytes. As a gene primarily functioning in lipid metabolism, INPP5 D participating in immune response by mainly maintaining the quantity of B lymphocytes and participating in phospholipid metabolism [53]. Cytohesin 1 interacting protein (CYTIP) encodes a protein containing 2 leucine zipper domains and a putative C-terminal nuclear targeting signal. Its major role is involved in the quantity maintenance, migration of leukocytes and lymphocytes [54].

The decreased above gene expression could explain the interference of normal immune response by TNT both *in vitro *and *in vivo*, which could lead to inflammation and other immune related toxicities caused by TNT.

### Network as a valuable approach for predicting *in vivo *function using *in vitro *data

Finally, not only could we find commonly regulated genes, functional terms, and pathways, but we were also able to identify conserved gene networks between *in vitro *and *in vivo *liver systems perturbed by TNT. There were two big conserved sub-networks, the TF PTTG1 centered gene network and the TFs PIR, BTBD11, HTATIP2 and CREG1-connected gene network (PBHC network). Interestingly, all the genes in the PTTG1 sub-network were associated with cell cycle function. For example, CDC2 and cyclin B (CCNB1) form a complex which is responsible for the onset of metaphase [55]. PTTG1, CDC2, and CCNB1 all participated in the mitotic roles of Polo-Like kinase pathway [56,57]. Furthermore, all the genes in the PTTG sub-network were downregulated by TNT *in vitro *and *in vivo*. Since cell cycle is the most significant functional term affected by TNT both *in vitro *and *in vivo *(Table 1), this conserved cell cycle relating network could well explain the common mechanism of TNT treated liver systems both *in vivo *and *in vitro*, which is that cell cycle progression is interfered by TNT, thereby leading to cell growth inhibition and cell death, ultimately cause toxicity [58-60]. We did observe liver cell death in rats exposed to high dose TNT (Deng et al., unpublished data).

In the PBHC sub-network, some genes such as CREG1, NTRK1, DUSP13, GSR, and TXNRD1 play a role in cell cycle and/or cell death. Many genes possess oxidoreductase activity [61], and are involved in oxidative stress process and play a crucial role in metabolizing toxic compounds and their metabolites. The TF PIR encodes a TF which is a member of the cupin superfamily. The encoded protein is a Fe(II)-containing nuclear protein expressed in all tissues of the body and concentrated within dot-like subnuclear structures [62]. Interactions with nuclear factor I/CCAAT box transcription factor as well as B cell lymphoma 3-encoded oncoprotein suggest the encoded protein may function as a transcriptional cofactor and be involved in the regulation of DNA transcription and replication. Interestingly, the TF HTATIP2 also has oxidoreductase activity [63], and connects two genes GSR and TXNRD1 that both possess oxidoreductase activity as well as both take part in NRF2-mediated oxidative stress pathway. HTATIP was reported to be regulated by NRF2, too [64].

Overall, we can see that the genes in the PBHC sub-network are involved in cell cycle growth, and/or oxidoreductase activity. Surprisingly, 17 genes in the PBHC sub-network (Figure 7) were all consistently upregulated by TNT both *in vivo *and *in vitro*. The induction of these gene expression lead to cell survival and removal of toxic products, therefore, this conserved sub-network may well explain the common detoxification mechanism both *in vivo *and *in vitro *liver systems treated with TNT.

We did not use only TNT-exposed samples but included all samples in the pool to construct static gene networks, which aim to uncover any gene interactions. In so doing, the more samples and conditions included make the network analyses more robust. For instance, Faith et al. (2007) used 445 arrays with over 180 various conditions to build an *E. Coli *static gene network. Because the genes we used are TNT-regulated genes, the constructed gene networks are specifically TNT-regulated networks.

We built separate gene networks using *in vitro *and *in vivo *samples and found commonly conserved networks. These common networks are ideal because they validate both *in vivo *and *in vitro *conditions.

## Conclusions

Our results indicate that TNT perturbed similar gene regulated networks when *in vitro *effects on hepatocyte cells are compared with *in vitro *effects on liver. Thus, gene regulatory networks obtained from an *in vitro *system can be predictive of *in vivo *function in the liver when perturbed by a chemical stressor such as TNT. Furthermore, PTTG1 regulated cell cycle may be a key targeted process indicator for TNT induced toxicity.

This study is the first report to use an *in vitro *transcriptional regulatory gene network to predict *in vivo *toxicity and mechanism induced by a chemical. Knowledge achieved from this innovative study can provide an efficient way to assess whether a soldier or civilian has been exposed to TNT and to find possible ways to prevent, treat, and reduce TNT induced adverse effects.

## Methods

### Microarray experimental design

Changes in gene expression were tested using Agilent commercial whole rat genome microarrays (4 X 44K). For *in vivo *experiment, one of five compounds (2,4-DNT, 2,6-DNT, TNT, 2-ADNT and 4-ADNT) were used to treat rats. Female Sprague-Dawley rats (175-225 grams) were from the in-house breeding colony (College of Pharmacy, University of Louisiana at Monroe [ULM] and treated in accordance with the *Guide for Use and Care of Animals *[65]. Breeders were from Harlan-Sprague Dawley in Madison, WI. Housing consisted of a 12 h light/dark cycle with *ad libitum *access to tap water and rodent chow (Harlan/Teklad 7012, Madison, WI). Rats were housed individually in polycarbonate cages on hardwood bedding (Sani-chips, Harlan/Tekland, Madison, WI) one week prior to treatment. Food was withdrawn the night before treatments, which were administered by gavage between 8 and 10 AM. Study protocols were preapproved by the ULM Animal Care and Use Committee.

Groups of rats were weighed and randomly assigned to treatment. Treatments were vehicle (5% v/v DMSO in corn oil), 2,4-DNT (4.8, 48, 96, and 192 mg/kg), 2A-DNT (4.4, 44, 87 and 174 mg/kg) and 4A-DNT (4.7, 47, 94 and 187 mg/kg), TNT (4.8, 48, 96 and 192 mg/kg), and 2,6-DNT (5.0, 25, 50 and 99 mg/kg). Rats were observed continuously for the first hour after dosing, hourly for 8 h and daily thereafter. Moribund rats were euthanized with CO2. Livers were excised and weighed. A portion of the liver was removed and placed in RNA Later (Ambion) following manufacturer's instruction and later used for genomic analyses. Remaining liver was flash frozen in liquid N_2 _and stored at -70°C for further analyses.

For *in vitro *experiment, primary cultured rat hepatocytes (Lonza, Walkersville, MD) were kept in an incubator at 37°C and 5% CO_2_. After seeding in flasks (3x10^6 ^cells/flask), the cells were treated with 10 mg/l TNT and vehicle control (DMSO). Three experimental replicates were used. After 24 h, the cells were isolated and lysed for RNA analysis. Cells were also treated with one of 106 compounds, including TNT (10 mg/l), and relative controls. At least three biological replicates were used for each unique condition (Additional file 3, Table S 3).

### Total RNA extraction

Total RNA was extracted from about 30 mg of liver tissue or cell pellet. Tissues or cells were homogenized in the lysis buffer with FAST Prep-24 from MP at speed 6.0/s twice, each last 30 s before using RNeasy kits (Qiagen). Total RNA concentrations were measured using NanoDrop^® ^ND-1000 Spectrophotometer (NanoDrop technologies, Wilmington, DE, USA). The integrity and quality of total RNA was checked on an Agilent 2100 Bioanalyzer (Palo Alto, CA). The gel-like images generated by the Bioanalyzer show that total RNAs have two bands, represent 18 S and 26 S RNA of mammalian RNA. Nuclease-free water (Ambion) was used to elute total RNA.

### Microarray hybridization

Rat whole genome oligo arrays in the format of 4X44K were purchased from Agilent. Sample cRNA synthesis, labeling, hybridization and microarray processing were performed according to manufacturer's protocol "One-Color Microarray-Based Gene Expression Analysis" (version 1.0). The Agilent One-Color Spike-Mix (part number 5188-5282) was diluted 5000-fold and 5 μl of the diluted spike-in mix was added to 1000 ng of each of the total RNA samples prior to labeling reactions. The labeling reactions were performed using the Agilent Low RNA Input Linear Amplification Kit in the presence of cyanine 3-CTP. The labeled cRNA from each labeling reaction was hybridized to individual arrays at 65°C for 17 h using the Agilent Gene Expression Hybridization Kit. After washing, the arrays were scanned at PMT levels 350 using GenePix 4200AL scanner (Molecular Device Inc.), the Feature extraction software (V. 9.5.1) from Agilent was used to automatically find and place microarray grids, reject outlier pixels, accurately determine feature intensities and ratios, flag outlier pixels, and calculate statistical confidences.

### Microarray data analysis

Microarray data analyses were processed with GeneSpring version 7.0 and 10.0. The sample quality control was based on the Pearson correlation of a sample with other samples in the whole experiment. If the average Pearson correlation with other samples was less than 80%, the sample was excluded for further analysis. If the scanned intensity was less than 5.0 for a probe, it was transformed to 5. A perchip (within) array normalization was performed using 50 percentile values of all the probe values in the array. Per gene (between) array normalization was also applied using the median value of a gene across all samples in the experiment. Probe features were first filtered using flags. A "present" or "absent" flag was defined using the Agilent *Feature Extraction 9.5.1 *software. Only a probe that had present flags in at least 50% samples of all the arrays was kept for further analyses. Data were subsequently log (base 2) transformed for statistical analyses.

To identify differentiated genes that responded to TNT treatment with various doses *in vivo*, One-Way ANOVA was performed across 5 doses at each time point (24 h or 48h). A cut off p value 0.05 was used to find statistically changed genes, and a 1.5 fold change showing at least one pair of doses at each time point was further applied to filter less significantly regulated genes. To idenitfy differential genes after TNT treatment *in vitro*, an un-paired t-test with cut off p value 0.05 was applied to compare control samples and TNT exposed samples. In additon, 1.5 fold change was applied to identify more signifcantly regulated genes.

### Gene functional analysis and pathway analysis

Significantly regulated probes were employed for two-way hierarchical clustering (clustering both genes and samples) using GeneSpring 7.0. A Pearson correlation with average linkage was applied for the clustering. Gene functional categories were classified according to the Ingenuity knowledge base tool. A Gene functional term enrichment p value less than 0.05 was considered significant. Pathway analysis was performed using the Ingenuity canonical pathways analysis tool. Similar to functional term analysis, a pathway with an enrichment p value less than 0.05 was considered to be a significantly regulated pathway.

### Reverse-transcription quantitative PCR (QRT-PCR)

Two-stage RT-QPCR were performed, 1000 ng of total RNA were first reverse transcribed into cDNA in a 20 μl reaction containing 250 ng random primers and SuperScript™ III reverse transcriptase (Invitrogen) following the manufacturer's instruction. The synthesized cDNA was diluted to 10 ng/μl as cDNA template. QPCR was performed on ABI Sequence Detector 7900. Each 20-μl reaction was run in duplicate and contained 6 μl (10ng/μl) of synthesized cDNA templates and 3 μl of nuclease-free water along with 1 μl of TaqMan gene specific assay and 10ul of 2× TaqMan universal PCR Master Mix (ABI). Cycling parameters were 95°C for 15 min to activate the DNA polymerase, then 40 cycles of 95°C for 15 s and 60°C for 1 min.

### Inferring gene networks using a reverse engineering algorithm

Gene networks were inferred using one of information-theoric network algorithm called Context Likelihood of Relatedness (CLR), which has been demonstrated to outperform other algorithms [66]. CLR computes a mutual information (MI) score between any pair of genes. The significance of a MI score was determined by comparing the MI score to a background distribution of MI values. This background distribution was achieved for each pair of genes with their MI scores with all other genes in the list. The CLR value between genes A and B was calculated by the formula:

$$\sqrt{Az^{2}+Bz^{2}}$$

*Az *and Bz are the *z*-score based on of A's and B's MI score distribution respectively between gene A and gene B [67]. Ten bins were used for the binning parameters of CLR, with a spline degree of 3. A cut-off *z*-score was set to 2.0 so that only the most significant interaction edges could be counted. Networks were visualized by Cytoscape [68,69].

## Authors' contributions

YD conceived and performed the study, analyzed the results, constructed the networks and drafted the manuscript. DRJ and CYA conducted the *in vitro *experiments. XG performed the microarray hybridization and QRT-PCR. JA processed the microarray data and helped for the network construction. EJP conceived and designed the study, coordinated the whole project. All authors read and approved the final manuscript.

## Supplementary Material

Additional file 1 **Table S1**. A table (supplementary table 1) providing a list of transcripts that were commonly regulated by TNT *in vivo *and *in vitro*. Click here for file

Additional file 2 **Table S2 and Figure S1**. A PDF including supplementary materials and methods, supplementary table 2 and supplementary Figure 1. Click here for file

Additional file 3 **Table S3**. A table (supplementary table 3) shows in vitro experimental design. Click here for file
