# Supplementary material for: In vitro gene regulatory networks predict in vivo function of liver
Source: BMC Syst Biol. 2010 Nov 12;4:153. doi: 10.1186/1752-0509-4-153 (PMC2998496; doi:10.1186/1752-0509-4-153)
Supplement: Additional file 2 — Table S2 and Figure S1. A PDF including supplementary materials and methods, supplementary table 2 and supplementary Figure 1. [file 1752-0509-4-153-S2.DOC]

**Supplementary materials and methods**

**1. Chemicals**

The chemical used for *in vivo* study including 2ADNT (99.9%) and 4ADNT (99.9%) that was obtained from SRI International (Menlo Park, CA). TNT (99.9%) was obtained from Eastman Chemical Company (Kingsport, TN). 2,4DNT (97%) and 2,6DNT (98%) was obtained from Sigma-Aldrich (St. Louis, MO).

The chemicals for the *in vitro* study were purchased from various sources: energetic chemicals including 2,4DNT, 2,6DNT, TNT, 2,4-diamino-6-nitrotoluene, 2-amino-4,4-DNT, 2ADNT, 4ADNT, CL-20, HMX, MNX, RDX,TNB, and TNX from Chem Service (West Chester, PA) and SRI International (Menlo Park, CA); remaining chemicals from Sigma-Aldrich (St. Louis, MO) and Fisher Scientific (Fair Lawn, NJ). The purity of all these compounds was equal or greater than 98%. Prior to testing, the compounds were made up and serially diluted in dimethyl sulfoxide, DMSO (Fisher Scientific, Fair Lawn, NJ). Metal compounds were made up and serially diluted in 0.1um-filtered, ultra-pure water before testing. The 24-h toxicity of each OMICS compound was first determined in the human hepatocyte cell line HepG2 using the Neutral Red cytotoxicity kit (In Vitro Toxicology Kit Tox-4) obtained from Sigma-Aldrich.

**2**. **Isolation of hepatocytes**. The primary rat hepatocytes, rtNHeps (AC-2630), isolated from male Sprague Dawley and its hepatocyte culture medium (HCM), and supplements and growth factors (CC-3198) were purchased from Cambrex BioScience (Walkersville, MD).

**3. Cell culture and Microarray experimental design**

The primary rtNHeps cells were reconstituted in HCM supplemented with ascorbic acid, fatty acid-free bovine serum albumin, transferrin, insulin, recombinant human epidermal growth factor, hydrocortisone 21 hemisuccinate, Gentamicin sulfate, and Amphotericin B immediately upon receipt. An aliquot of the cell suspension was stained in a 0.05% Trypan Blue solution and counted under an inverted microscope. The cells were then seeded at 3 x 10^6^ cells per (Type 1 collagen-coated) T-75 flask. The flasks were left in a 37C, 5% CO_2_ incubator overnight to allow for cell attachment.

The cells were replenished with fresh HCM and dosed in triplicate flasks with the non-toxic concentration of each compound (pre-determined in the HepG2 cells) at 1% DMSO (v/v) or with solution at 1% water (v/v). Another set of triplicate flasks were dosed with the appropriate solvent control. Hence, for every 3 chemicals and a solvent control, a total of 12 flasks were used. There are 99 compounds in total were done in 33 sets. For 11 compounds dosage study, each chemical has 3 treatments plus control, all have 3 biological replicateChanges in gene expression were tested using Agilent commercial whole rat genome microarrays (4 X 44K). For in vitro experiment, primary cultured rat hepatocytes were treated one of 105 compounds with relative controls. At least three biological replicates were used for each unique condition. The dose used for TNT in vitro was 10 ppm.

**4. Animals and Treatment:** Female Sprague-Dawley rats (175-225 grams) were from the in-house breeding colony (College of Pharmacy, University of Louisiana at Monroe [ULM]) and treated in accordance with the *Guide for Use and Care of Animals* (National Research Council 1996). Breeders were from Harlan-Sprague Dawley in Madison, WI. Housing consisted of a 12 h light/dark cycle with *ad libitum* access to tap water and rodent chow (Harlan/Teklad 7012, Madison, WI). Rats were housed individually in polycarbonate cages on hardwood bedding (Sani-chips, Harlan/Tekland, Madison, WI) one week prior to treatment. Food was withdrawn the night before treatments, which were administered by gavage between 8 and 10 AM. Study protocols were preapproved by the ULM Animal Care and Use Committee.

**Supplementary figures:**

**Fig. 1. Quantitative real-time RT-PCR (QRT-PCR) verification of microarray gene expression.** 13 genes were selected to validate the microarray results. Both QRT-PCR and microarray results of the genes ABCC3and AKR7A3 (A), CCNB1 and CCND2 (B), CREG1 and CXCL12 (C), CYP1A1 and GSTP1(D), LGALS1 and NQO1 (E), PIR and PPARG(F) were presented. The experimental design was the same as the microarray experiment. The expression value for a given gene was represented as a log2 ratio of ratio of exposed versus respective control RNA. For a gene, the upper panel is microarray result and the lower panel is QRT-PCR microarray result.

Fig. 1A


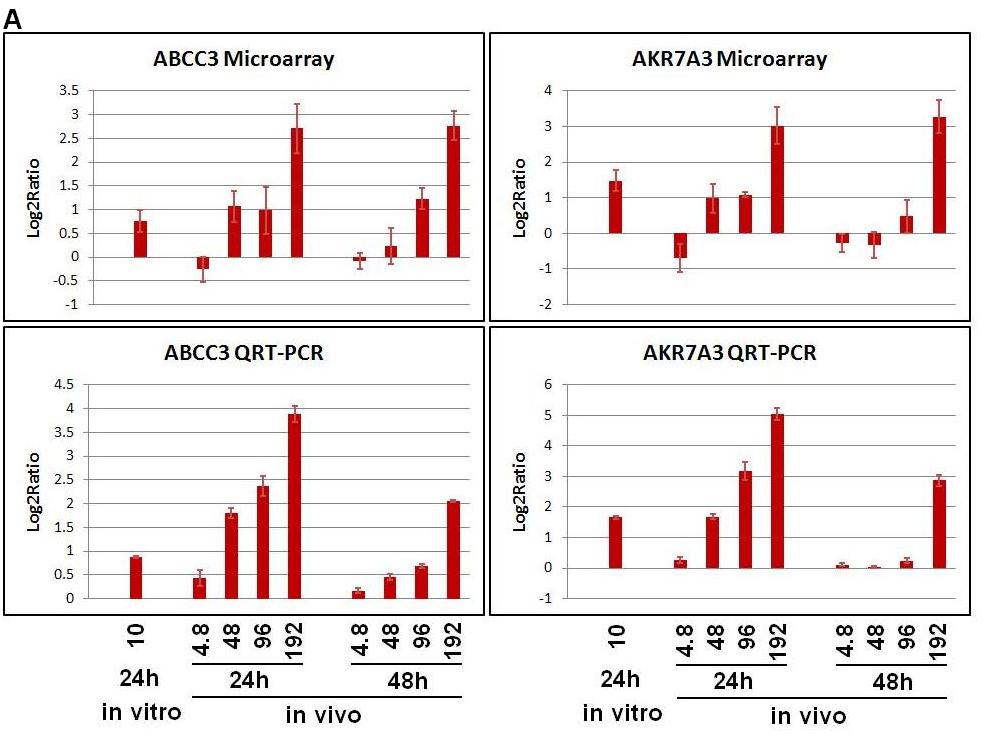


Fig. 1B


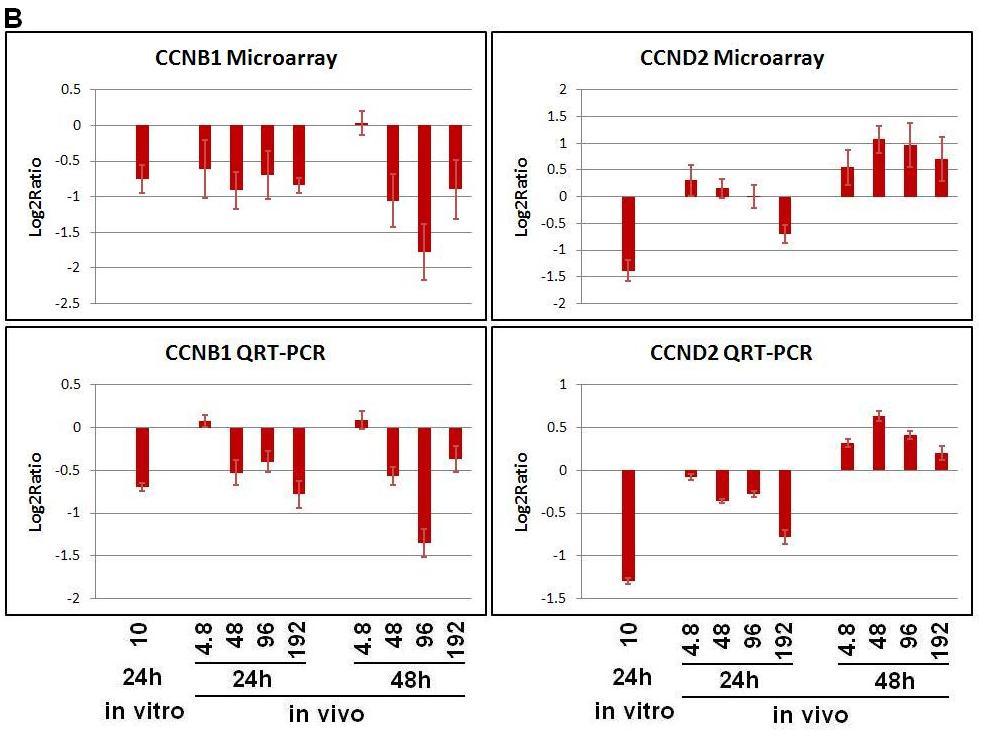


Fig. 1C


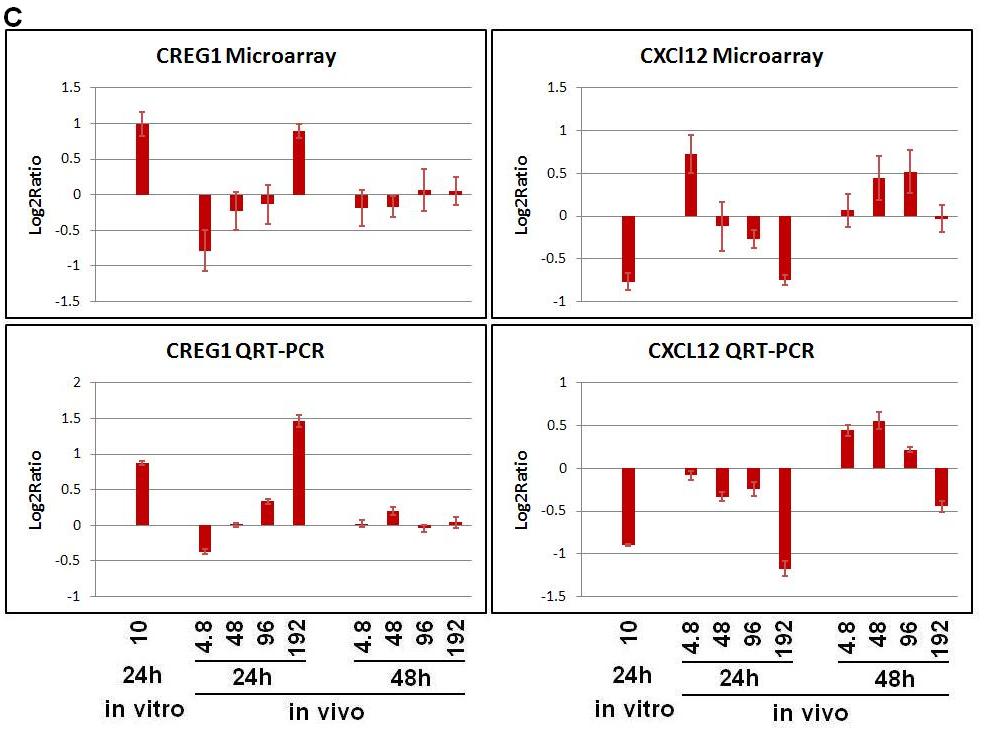


Fig. 1D


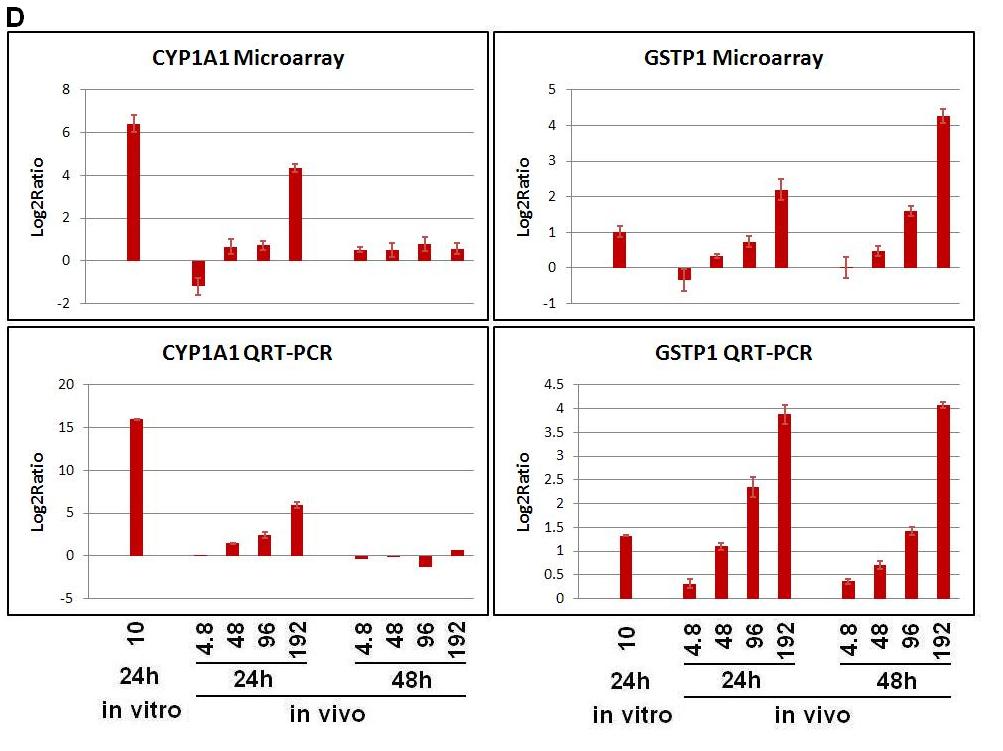


Fig. 1E


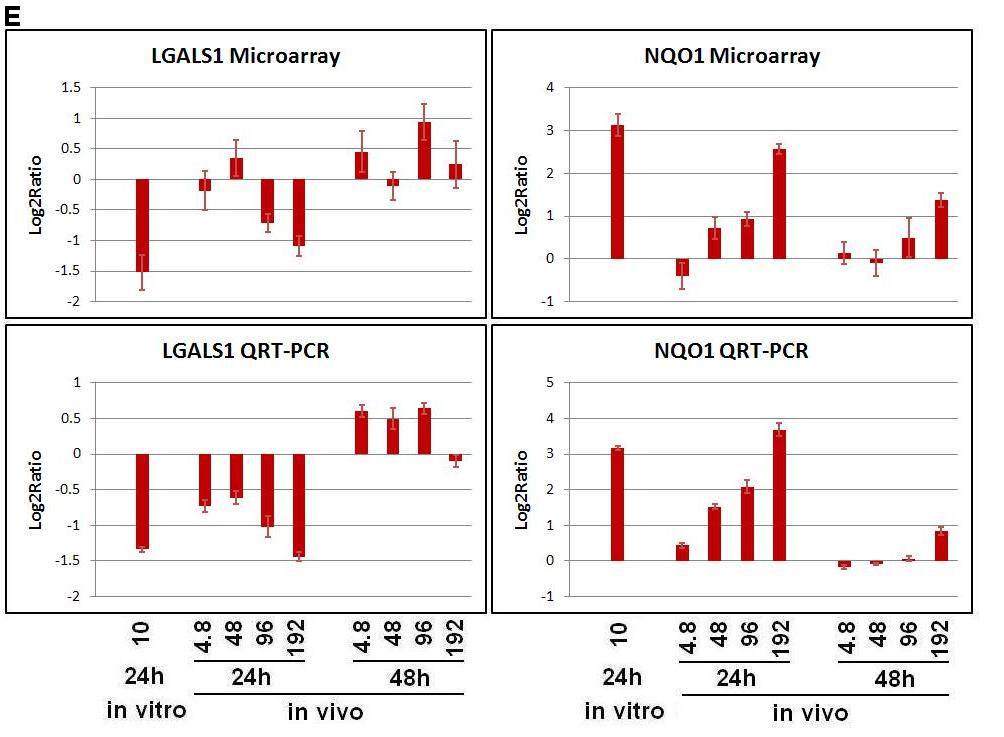


Fig. 1F


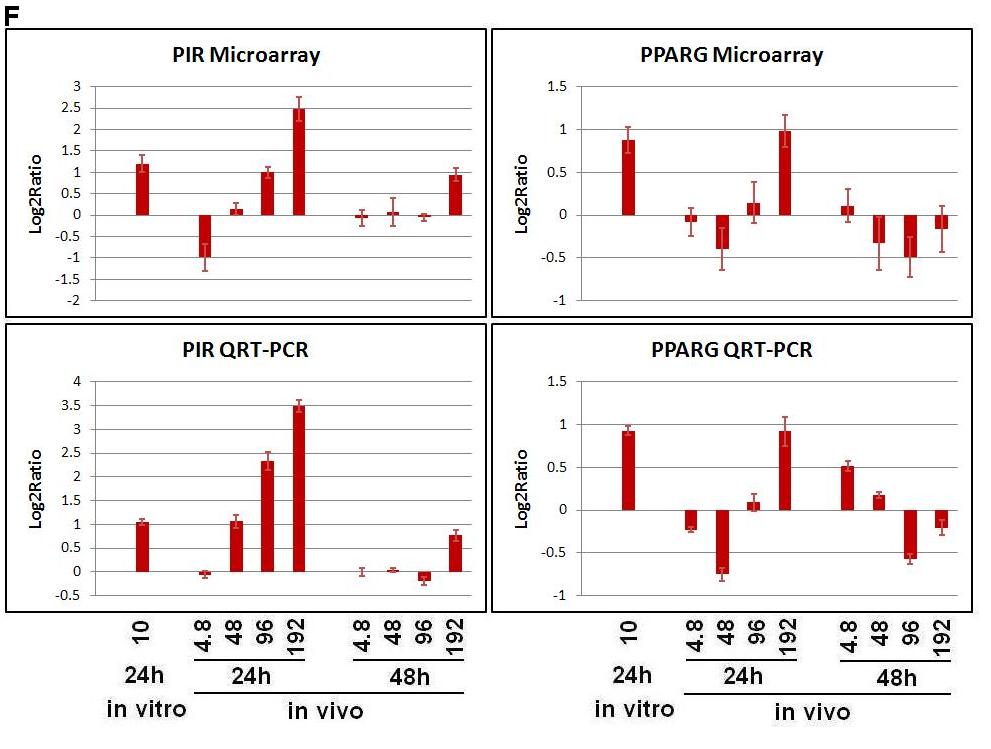


| Chemicals | Time | Doses (mg/kg) | Biological replicates for each condition |
| --- | --- | --- | --- |
| 2,4DNT | 24h | 0, 4.98, 49.8, 99.5, 199 | 4 |
|  | 48h |  |  |
| 2,6DNT | 24h | 0, 5, 25, 50, 99 | 4 |
|  | 48h |  |  |
| 2ADNT | 24h | 0. 4.4, 44, 87, 174 | 4 |
|  | 48h |  |  |
| 4ADNT | 24h | 0, 4.7, 47, 93, 187 | 4 |
|  | 48h |  |  |
| TNT | 24h | 0, 4.8, 48, 96, 192 | 3-4 |
|  | 48h |  |  |

Supplementary table 2 in vivo experimental design
